# Effects of lime and oxalic acid on antioxidant enzymes and active components of *Panax notoginseng* under cadmium stress

**DOI:** 10.1038/s41598-022-15280-w

**Published:** 2022-07-06

**Authors:** Qi Li, Na Jiang, Xinyue Mei, Yanqun Zu, Zuran Li, Li Qin, Bo Li

**Affiliations:** 1 College of Resources and Environment, Yunnan Agricultural University, Kunming, 650201 China; 2 College of Animal Science and Technology, Yunnan Agricultural University, Kunming, 650201 China; 3 College of Landscape and Horticulture, Yunnan Agricultural University, Kunming, 650201 China

**Keywords:** Biophysics, Ecology, Physiology, Ecology, Environmental sciences

## Abstract

Cadmium (Cd) pollution poses potential safety risks for *Panax notoginseng* cultivation, a medicinal plant in Yunnan. Under exogenous Cd stress, field experiments were conducted to understand the effects of lime (0, 750, 2250 and 3750 kg hm^−2^) applied and oxalic acid (0, 0.1 and 0.2 mol L^−1^) leaves sprayed on Cd accumulation, antioxidant system and medicinal components of *P. notoginseng*. The results showed that Lime and foliar spray of oxalic acid were able to elevate Ca^2+^ and alleviate Cd^2+^ toxicity in *P. notoginseng* under Cd stress. The addition of lime and oxalic acid increased the activities of antioxidant enzymes and alters osmoregulator metabolism. The most significant increase in CAT activities increased by 2.77 folds. And the highest increase of SOD activities was 1.78 folds under the application of oxalic acid. While MDA content decreased by 58.38%. There were very significant correlation with soluble sugar, free amino acid, proline and soluble protein. Lime and oxalic acid were able to increase calcium ions (Ca^2+^), decrease Cd content and improve the stress resistance of *P. notoginseng*, while increasing the production of total saponins and flavonoids. Cd content were the lowest, 68.57% lower than controls, and met the standard value (Cd ≤ 0.5 mg kg^−1^, GB/T 19086-2008). The proportion of SPN was 7.73%, which reached the highest level of all treatments, the flavonoids content increased significantly by 21.74%, which reached the medicinal standard value and optimal yield.

## Introduction

As a common pollution element in cultivated soil, cadmium (Cd) is easy to migrate and has significant biological toxicity^1^. El-Shafey et al.^2^ reported that Cd toxicity affects the quality and productivity of plants to be used. In recent years, Cd exceedance in arable soils in the southwest has been very serious. Yunnan Province is the kingdom of biodiversity in China, in which the species of medicinal plants rank first in the country. However, the abundance of mineral resources in Yunnan Province has led to the inevitable heavy metal contamination of soil during mining affecting the production of local medicinal plants.

*Panax notoginseng* (Burkill) Chen^3^ is a very precious perennial herb medicinal plant, which is belonging to *Panax ginseng* genus of Araliaceae^4^. The root of *P. notoginseng* has the efficacy of removing stasis and eliminating pain by invigorating blood circulation. The main producing area is in Wenshan Prefecture, Yunnan Province^5^. Cd contamination was present in more than 75% of the soil area of the local *P. notoginseng* planting area, and in different parts more than 81–100%^6^. The toxic effect of Cd also greatly reduced the yield of medicinal components of *P. notoginseng*, especially saponins and flavonoids. Saponins, a class of glycosides in which aglycones are triterpenoids or spirostanoids, are the main active ingredients of many Chinese herbs and contain saponins. Some saponins also have antibacterial activity or antipyretic, sedative, anticancer and other valuable biological activities^7^. Flavonoids, broadly referring to a series of compounds in which the two phenyl rings with a phenolic hydroxyl group are linked to each other by a central three carbon atom, the basic parent nucleus of which is 2-phenylchromanone^8^. It is a strong antioxidant that effectively scavenges oxygen free radicals in plants, also can inhibit the exudation of inflammatory biological enzymes, improve wound healing and analgesia, reduce cholesterol, etc., is one of the main active ingredients of *P. notoginseng*^9^. It is quite necessary to solve the problem of soil Cd pollution in *P. notoginseng* growing regions to guarantee the yield of its main medicinal components.

Lime is one of the commonly used passivation agents for in-situ fixed remediation of soil Cd contamination^10^. It affects the adsorption and precipitation of Cd in soil, and reduces the bioavailability of Cd in soil by increasing pH and changing soil cation exchange capacity (CEC), soil salt saturation (BS), soil redox potential (Eh)^3,11^. In addition, lime provides a large amount of Ca^2+^, forms ionic antagonism with Cd^2+^, competing for root adsorption sites, preventing the transport of Cd to shoot, and low biological toxicity. When 50 mmol L^−1^ Ca added under Cd stress, the transport of Cd in *Sesamum indicum* L. leaves was inhibited, and the accumulation of Cd decreased by 80%. A large number of related studies were reported in rice (*Oryza sativa* L.) and other crops^12,13^.

Foliar spraying of crops to regulate the accumulation of heavy metals is a new method for heavy metal control in recent years. The principles are mainly related to chelating reaction in plant cells, precipitate heavy metals on the cell wall, and inhibit the absorption of heavy metals by plants^14,15^. Oxalic acid, as a stable dicarboxylic acid chelating agent, can directly chelate heavy metal ions in plants to alleviate toxicity. Studies showed that oxalic acid in soybean can chelate Cd^2+^, and expel Cd-containing crystals through apical cells of trichomes, reduce Cd^2+^ content in vivo^16^. Oxalic acid could regulate soil pH and enhance the activities of superoxide dismutase (SOD), peroxidase(POD) and catalase (CAT), regulate the metabolism of osmotic regulators such as soluble sugar, soluble protein, free amino acid and proline^17,18^.Oxalic acid and excess Ca^2+^ in plants form calcium oxalate precipitates under the action of nucleating proteins. Regulating the concentration of Ca^2+^ in plants can effectively ensure the regulation of dissolved oxalic acid and Ca^2+^ in plants, and avoid excessive accumulation of oxalic acid and Ca^2+^^19,20^.

The amount of lime applied is one of the key factors affecting the remediation effect. It was found that the lime application ranged from 750 to 6000 kg hm^−2^. For acid soils with pH5.0–5.5, the effect of lime application rate of 3000–6000 kg hm^−2^ was significantly higher than that of 750 kg hm^−2^^21^. But excessive application of lime will cause some negative effects on soil, such as large changes in soil pH, soil compaction, and so on^22^. As a consequence, we make the treatment level of CaO is 0, 750, 2250 and 3750 kg hm^−2^. When oxalic acid was applied to Arabidopsis, it was found that Ca^2+^ decreased significantly at the concentration of 10 mmol L^−1^, and the CRT gene family affecting Ca^2+^ signal transduction responded strongly^20^. And the accumulation of some previous studies let us determine the concentration of this test and continue to explore the interaction effect of exogenous additives on Ca^2+^ and Cd^2+^^23–25^. Therefore, we aimed to investigate the regulatory mechanisms underlying the effects of exogenously applied lime and foliar spray of oxalic acid on the Cd content and stress resistance of *P. notoginseng* in Cd contaminated soils, and to further explore better ways to guarantee the medicinal quality and yield of *P. notoginseng*. It provides valuable information that is worth guiding the process of enhancing the cultivation scale of herbaceous plants in Cd contaminated soils, and ensures high-quality, sustainable production in market requirements for medicinal products.

## Materials and methods

### Experimental design

The local cultivar Wenshan *P. notoginseng* was used as material, the field experiment was carried out in Lannizhai, Qiubei County, Wenshan Prefecture, Yunnan Province (N 24°11′, E 104°3′, 1 446 m above sea level). The average annual temperature was 17 °C and the annual average precipitation was 1250 mm. The background value of the tested soil was TN 0.57 g kg^−1^, TP 1.64 g kg^−1^, TK 16.31 g kg^−1^, OM 31.86 g kg^−1^, alkali hydrolyzed N 88.82 mg kg^−1^, available P 18.55 mg kg^−1^, available K 100.37 mg kg^−1^, total Cd 0.3 mg kg^−1^ and pH 5.4.

The 6 mg kg^−1^ Cd^2+^ (CdCl_2_·2.5H_2_O) and the lime treatments (0, 750, 2250 and 3750 kg hm^−2^) were applied and mixed with the surface 0–10 cm soil layer of each plot on December 10, 2017. Each treatment was repeated for 3 times. The experimental plots were arranged randomly, and the area of each plot was 3 m^2^. One-year-old *P. notoginseng* seedlings were transplanted after 15 days of soil culture. The shading net was used and the light intensity of *P. notoginseng* in the shading shed was about 18% of the normal natural light intensity. It was cultivated according to the local conventional cultivation and management. Until the maturity period of *P. notoginseng* in 2019, oxalic acid was sprayed in the form of sodium oxalate. The concentrations of oxalic acid were 0, 0.1 and 0.2 mol L^−1^, and the pH was adjusted to 5.16 with NaOH, which was simulated the average pH of litter leaching solution. The upper and lower surfaces of leaves were sprayed at 8:00 am in the morning once a week. After 4 times spray, 3-year-old *P. notoginseng* plants were collected in the 5th week.

### Sampling and pretreatment

The 3-year-old *P. notoginseng* plants sprayed with oxalic acid were collected in the field in November 2019. Part of the 3-year-old *P. notoginseng* plants samples which needed to determine physiological metabolism and enzyme activity, were put into a frozen tube, quick-frozen with liquid nitrogen, and then transferred to the refrigerator at − 80 °C. Part of the root samples, which need to determine the contents of Cd and active components in the mature stage, were dried 30 min at 105 °C after washing with tap water and to constant weight at 75 °C, then grind samples in a mortar for preservation.

### Parameters determination

#### Cadmium contents

Some 0.2 g of dried plant samples were weighed and put into triangular flask, add 8 mL of HNO_3_ and 2 mL HClO_4_, and seal overnight. The next day was digested by electrothermal with a curved neck funnel placed in triangular bottles until a white smoke appeared and the digestive solution to clarify. After cooling to room temperature, the mixture was moved to 10 ml volumetric flask. The content of Cd was determined by atomic absorption spectrometer (Thermo ICE™ 3300 AAS, USA). (GB/T 23739-2009).

#### Calcium contents

Some 0.2 g of dried plant samples were weighed and put into 50 mL plastic bottle, add 1 mol L^−1^ HCL 10 mL, sealed and shaken for 15 h, then filtered. The pipette absorbs the desired amount of filtrate to properly dilute, and add SrCl_2_ solution to make the concentration of Sr^2+^ 1 g L^−1^. The content of Ca was determined by atomic absorption spectrometer (Thermo ICE™ 3300 AAS, USA).

#### MDA contents and antioxidase activities

The malondialdehyde (MDA), superoxide dismutase (SOD), peroxidase (POD) and catalase (CAT) were determined by the corresponding kit with reference to the kit method (DNM-9602, Beijing Pulang New Technology Co., Ltd., Product registration number: Beijing Pharmaceutical Dianji (quasi) Word No. 2400147 of 2013).

#### Soluble sugar contents

Some 0.05 g of *P. notoginseng* sample was weighed and added anthrone-sulfuric acid reagent along the test tube wall. The test tube was shook 2–3 s to make the liquid was fully mixed evenly. The test tube was placed on the test tube rack to develop color 15 min. The soluble sugar content was determined with UV–visible spectrophotometry (UV-5800, Shanghai Yuanxi Instrument Co. Ltd., China) at the 620 nm wavelength^26^.

#### Soluble protein contents

Some 0.5 g *P. notoginseng* fresh sample was weighed and ground to the homogenate with 5 mL distilled water, then centrifuged 10,000 g for 10 min. The supernatant was diluted to the fixed volume. The Coomassie brilliant blue method was used. The soluble protein contents were determined with UV–visible spectrophotometry (UV-5800, Shanghai Yuanxi Instrument Co. Ltd., China) at the 595 nm wavelength and calculated from the bovine serum albumin standard curve^27^.

#### Free amino acid contents

Some 0.5 g fresh sample was weighed and 5 ml 10% acetic acid was added to grind to homogenate, then filtered and diluted to a constant volume. The ninhydrin solution coloration method was used. The free amino acid contents were determined with UV–visible spectrophotometry (UV-5800, Shanghai Yuanxi Instrument Co. Ltd., China) at 570 nm wavelength, and calculated from leucine standard curve^28^.

#### Proline contents

Some 0.5 g fresh sample was weighed and 5 mL 3% sulfosalicylic acid solution was added, then heating in water bath and shaking for 10 min. After cooling, the solution was filtered and diluted to a constant volume. The acid ninhydrin coloration method was used. The proline contents were determined with UV–visible spectrophotometry (UV-5800, Shanghai Yuanxi Instrument Co. Ltd., China) at 520 nm wavelength, and calculated by proline standard curve^29^.

#### Saponins contents

The contents of saponins were determined by High Performance Liquid Chromatography (HPLC), referring to the Pharmacopoeia of the people’s Republic of China (2015 Edition). The basic principle of HPLC is to take the liquid under high pressure as the mobile phase and adopt the separation technology of very fine particle high-efficiency stationary phase column chromatography. The operating techniques are as follows:

1. HPLC conditions and system applicability test (Table 1): Gradient elution was carried out according to the following table by using octadecylsilane bonded silica gel as filler, acetonitrile as mobile phase A and water as mobile phase B. The detection wavelength was 203 nm. The number of theoretical plates should not be less than 4000 according to the R1 peak of *P. notoginseng* saponins.
2. Preparation of reference solution: ginsenoside Rg1, ginsenoside Rb1, and notoginsenoside R1 were precisely weighed and methanol was added to prepare the mixed solution containing ginsenoside Rg1 0.4 mg, ginsenoside Rb1 0.4 mg, and notoginsenoside R1 0.1 mg per 1 mL.
3. Preparation of test solution: 0.6 g *P. notogensing* powder was weighed, and 50 mL methanol was dded. The mixed solution was weight (W1) and placed it overnight. Then the mixed solution was kept in lightly boiled in a water bath at 80 °C for 2 h. After cooling, the mixed solution was weighed, and added made methanol to the first weight W1. After then shook well, it was filtered. The filtrate solution was kept for determination.
4. The saponins contents were determined with 10 μL of the reference solution and 10 μL of the filtrate solution precisely absorbed and injected into the HPLC (Thermo HPLC-ultimate 3000, Seymour Fisher Technology Co., Ltd.)^24^.
5. Standard curve: The mixed standard solution of Rg1, Rb1 and R1 are determined with the chromatographic conditions the same as above. The measured peak area is ordinate and the concentration of saponins in the standard solution is abscissa for the standard curve calculated. The measured peak area of sample is substituted into the standard curve to calculate the concentration of saponins.

**Table 1:** Gradient elution of the mobile phases.

Time (min)	Mobile phase A (%)	Mobile phase B (%)
1–6	20 → 30	80 → 70
6–14	30 → 40	70 → 60
14–20	40 → 30	60 → 70
20–25	30 → 20	70 → 80
25–35	20	80

#### Flavonoids contents

0.1 g of *P. notogensings* samples were weighted and the 50 mL 70%CH_3_OH solution was added. The ultrasonic extraction was conducted for 2 h, then centrifuged under 4000 r min^−1^ for 10 min. 1 mL the supernatant was took out and diluted 12 times. The content of flavonoids was determined with UV–visible spectrophotometry (UV-5800, Shanghai Yuanxi Instrument Co. Ltd., China) at 249 nm wavelength. Quercetin was one of standard common substances^8^.

### Statistical analysis

The data were sorted out by Excel 2010 software. Analysis of variance of data was evaluated by SPSS statistical 20 software. And the pictures were plotted by origin Pro 9.1. Statistical values that were calculated include mean ± SD. The statement of statistical significance was based on *P* < 0.05.

## Results

### Contents of Cd and Ca in Panax notogensing roots

The Ca content of *P. notoginseng* roots increased significantly with the increase of lime application rates under the same concentration of oxalic acid sprayed on leaves (Table 2). Compared with no lime application, the Ca content was the highest increased by 212% under 3750 kg hm^−2^ lime without spraying oxalic acid. The content of Ca slightly increased with the increase of oxalic acid spraying concentrations under the same rate of lime application.

**Table 2:** Effects of foliar spraying of oxalic acid on contents of Cd and Ca in roots of *Panax notoginseng* under Cd stress.

Content (mg kg^−1^)	Lime application (kg hm^−2^)	Oxalic acid concentration (mol L^−1^)		
		0.0	0.1	0.2
Ca	0	0.75 ± 0.07c	0.76 ± 0.25c	0.67 ± 0.20c
	750	1.66 ± 0.29b	1.41 ± 0.07b	1.66 ± 0.15b
	2250	2.11 ± 0.24ab	2.17 ± 0.10a	1.73 ± 0.11b
	3750	2.34 ± 0.16a	1.92 ± 0.14a	2.12 ± 0.08a
Cd	0	0.70 ± 0.09a	0.61 ± 0.07a	0.56 ± 0.07a
	750	0.47 ± 0.04b	0.33 ± 0.04b	0.28 ± 0.01b
	2250	0.31 ± 0.03c	0.22 ± 0.05c	0.23 ± 0.03b
	3750	0.23 ± 0.04c	0.25 ± 0.01bc	0.27 ± 0.02b

Date were means ± SD. Different lowercase letters in the same column indicate significant differences at the level of *P* < 0.05.

The contents of Cd in roots ranged from 0.22 to 0.70 mg kg^−1^. The content of 2250 kg hm^−2^ Cd decreased greatly with the increase of lime application rates under the same spraying concentration of oxalic acid. Compared with the control, the root Cd contents decreased by 68.57% under the application of 2250 kg hm^−2^ lime and 0.1 mol L^−1^ oxalic acid spraying. The Cd contents of *P. notoginseng* roots decreased significantly with the increase of oxalic acid spraying concentrations under application of non-lime and 750 kg hm^−2^ lime. The root Cd contents decreased at first and then increased with the increase of oxalic acid concentrations under the application of 2250 kg hm^−2^ lime and 3750 kg hm^−2^ lime. In addition, the Bivariate analysis showed that the Ca content of *P. notoginseng* roots was significantly affected by lime (F = 82.84**), and the Cd content of *P. notoginseng* roots was significantly affected by lime (F = 74.99**) and oxalic acid (F = 7.72*).

### MDA contents and relative antioxidase activities

**Figure 1:**
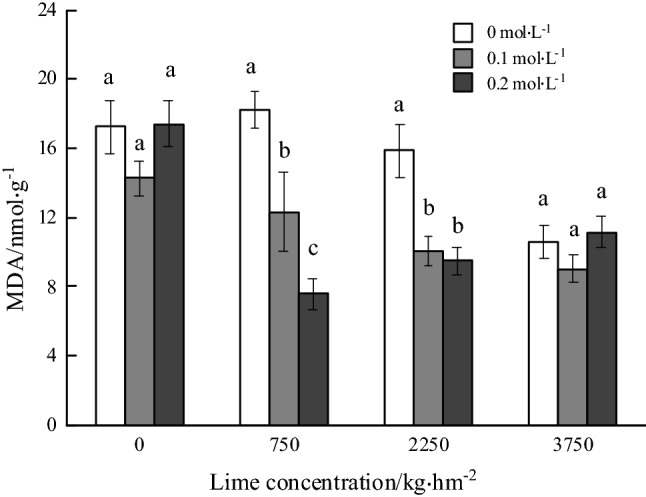
Effects of foliar spraying of oxalic acid on contents of malondialdehyde in roots of *Panax notoginseng* under Cd stress. *Notes* The figure legend showed the spray concentration of oxalic acid (mol L^−1^), different lowercase letters indicate significant differences between treatments at the same lime application rate (*P* < 0.05). The same below.

The content of MDA decreased greatly with the increase of the rates of lime application and oxalic acid spraying concentrations. There was no significant difference in the content of MDA in the roots of *P. notoginseng* with non-lime and 3750 kg hm^−2^ lime application. Under 750 kg hm^−2^, 2250 kg hm^−2^ lime application, the MDA content with 0.2 mol L^−1^ oxalic acid spraying concentration treatment decreased by 58.38% and 40.21% comparing with non-oxalic acid spraying application, respectively. The content of MDA (7.57 nmol g^−1^) was the lowest under 750 kg hm^−2^ lime application and 0.2 mol L^−1^ oxalic acid spraying treatment (Fig. 1).

**Figure 2:**
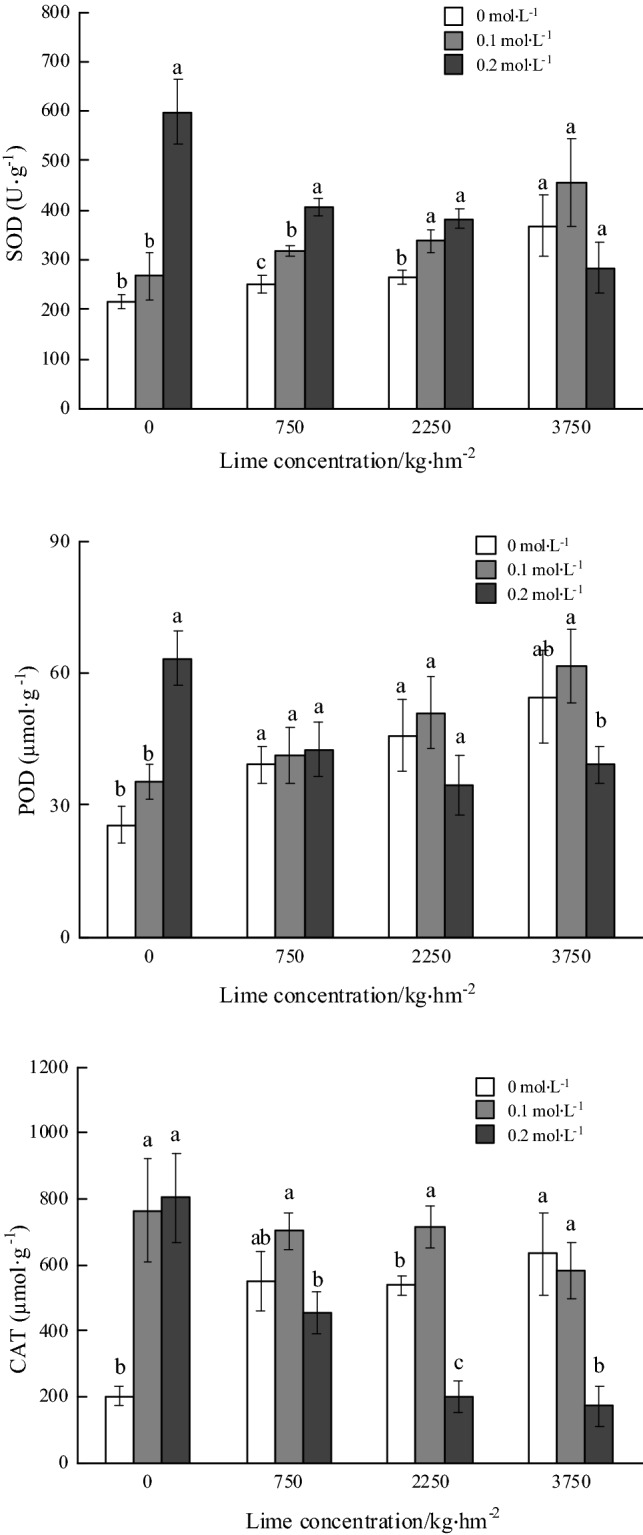
Effects of foliar spraying of oxalic acid on activities of superoxide dismutase, peroxidase and catalase in roots of *Panax notoginseng* under Cd stress.

There was no significant difference in SOD activity in the roots of *P. notoginseng* except for the application rate of 3750 kg hm^−2^ lime. Under 0, 750 and 2250 kg hm^−2^ lime application, the SOD activities of 0.2 mol L^−1^ oxalic acid spraying treatment were significantly higher than that without oxalic acid application, which increased by 177.89%, 61.62% and 45.08%, respectively. The SOD activity (598.18 U g^−1^) in roots was the highest non-lime application and 0.2 mol L^−1^ oxalic acid spraying treatment. The SOD activities increased with the increase of lime application rates with the same concentration of no oxalic acid or 0.1 mol L^−1^ oxalic acid spraying treatment. The SOD activities decreased significantly with 0.2 mol L^−1^ oxalic acid spraying treatment (Fig. 2).

The similar as the root SOD activity, the POD activity (63.33 μmol g^−1^) in the root was the highest with non-lime and 0.2 mol L^−1^ oxalic acid spraying treatment, which was 148.35% higher than that of the control (25.50 μmol g^−1^). The POD activity increased at first and then decreased with the increase of oxalic acid spraying concentration and 3750 kg hm^−2^ lime application treatment. The POD activity decreased by 36.31% with 0.2 mol L^−1^ oxalic acid treatment compared with 0.1 mol L^−1^ oxalic acid treatment (Fig. 2).

The CAT activities were significantly higher than that of the control except for 0.2 mol L^−1^ oxalic acid spraying and 2250 kg hm^−2^ or 3750 kg hm^−2^ lime application treatments. The CAT activities of increased by 276.08%, 276.69% and 33.05% with 0.1 mol L^−1^ oxalic acid spraying and 0, 2250 kg hm^−2^ or 3750 kg hm^−2^ lime application treatments, respectively, compared with that without oxalic acid spraying. The root CAT activity (803.52 μmoL g^−1^) was the highest with non-lime and 0.2 mol L^−1^ oxalic acid treatment. The CAT activity (172.88 μmol g^−1^) was the lowest with 3750 kg hm^−2^ lime and 0.2 mol L^−1^ oxalic acid treatment (Fig. 2).

Bivariate analysis showed that the CAT activity and MDA of *P. notoginseng* roots was significantly relationship with the amount of oxalic acid spraying or lime application and both treatment (Table 3). The root SOD activity was significantly relationship with both of lime and oxalic acid treatment or oxalic acid spraying concentration. The activity of POD in roots was significantly relationship with the lime application rate or both of lime and oxalic acid treatment.

**Table 3:** Variance analysis of the effects of oxalic acid, calcium and cadmium on antioxidant enzyme activity and the contents of malondialdehyde in the roots of *Panax notoginseng* (F value).

Treatments	CAT	MDA	SOD	POD
Lime	3.07*	25.88**	1.59	3.25*
Oxalic acid	22.63**	29.14**	21.71**	1.59
Lime × Oxalic acid	15.22**	10.14**	14.08**	8.03**

In the two-way analysis of variance, * indicates *P* < 0.05, and ** indicates *P* < 0.01.

### Contents of medicals components

#### Contents of soluble sugar and soluble protein

**Figure 3:**
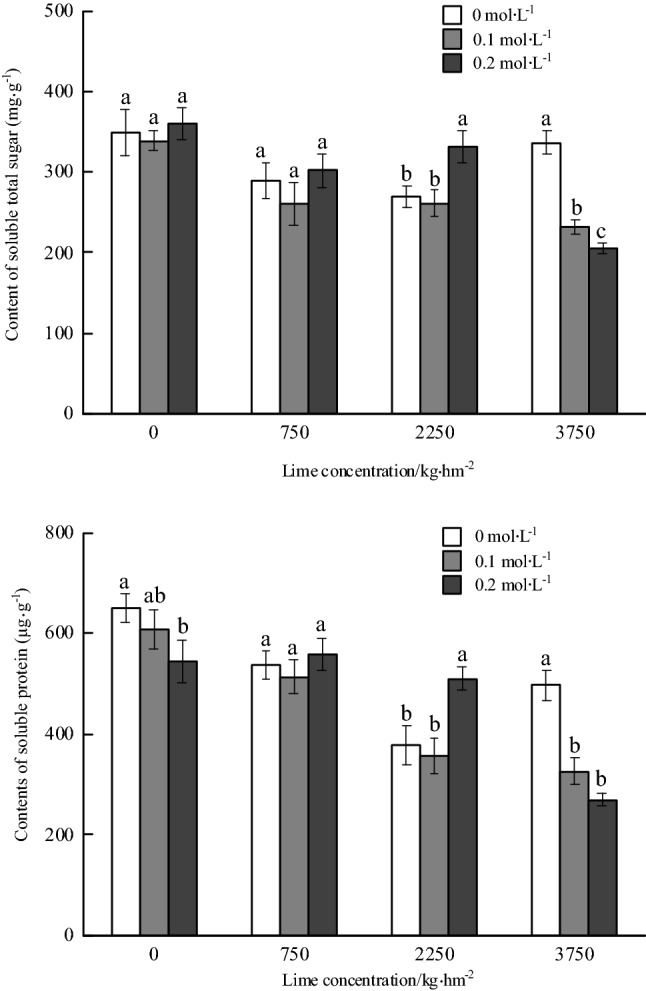
Effects of foliar spraying of oxalic acid on contents of soluble total sugar and soluble protein in the roots of *Panax notoginseng* under Cd stress.

The content of soluble sugar in roots decreased with the increase of the rate of lime application and oxalic acid spraying concentration. There was no significant difference in the content of soluble sugar in the roots of *P. notoginseng* under without lime and 750 kg hm^−2^ lime application. Under 2250 kg hm^−2^ lime application, the soluble sugar content with 0.2 mol L^−1^ oxalic acid treatment was significantly higher than that of non-oxalic acid spraying, which increased by 22.81%. Under the application of lime 3750 kg hm^−2^, the soluble sugar content decreased significantly with the increase of oxalic acid spraying concentration. The soluble sugar content with 0.2 mol L^−1^ oxalic acid spraying treatment decreased by 38.77% compared with that of non-oxalic acid spraying. Moreover, the soluble sugar content with 0.2 mol L^−1^ oxalic acid spraying treatment was the lowest, which was 205.80 mg g^−1^ (Fig. 3).

The content of soluble protein in roots decreased with the increase of the amount of lime application and oxalic acid spraying treatment. In the absence of lime, the soluble protein content with 0.2 mol L^−1^ oxalic acid spraying treatment was significantly lower 16.20% than that of the control. Under 750 kg hm^−2^ lime application, there was no significant difference in the content of soluble protein in the roots of *P. notoginseng*. Under 2250 kg hm^−2^ lime application, the soluble protein content with 0.2 mol L^−1^ oxalic acid spraying treatment was significantly higher 35.11% than that of non-oxalic acid spraying. Under the application of lime 3750 kg hm^−2^, the soluble protein content decreased significantly with the increase of oxalic acid spraying concentration, and the soluble protein content (269.84 μg g^−1^) was the lowest under 0.2 mol L^−1^ oxalic acid spraying treatment (Fig. 3).

#### Contents of free amino acid and proline

**Figure 4:**
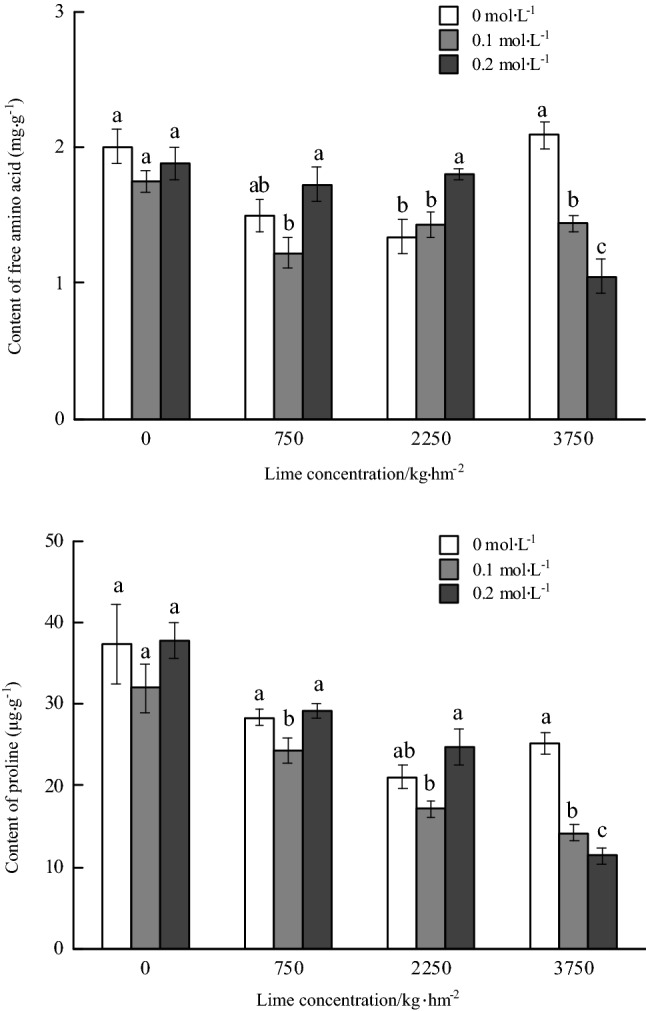
Effects of foliar spraying of oxalic acid on contents of free amino acid and proline in the roots of *Panax notoginseng* under Cd stress.

There was no significant difference in the content of free amino acid in the roots of *P. notoginseng* under without lime application. The content of free amino acid decreased at first and then increased with the increase of oxalic acid spraying concentrations and 750 kg hm^−2^ lime application. The content of free amino acid significantly increased by 33.58% with 2250 kg hm^−2^ lime application and 0.2 mol L^−1^ oxalic acid spraying treatment compared with non-oxalic acid spraying treatment. The content of free amino acid decreased significantly with the increase of the concentration of oxalic acid spraying and the application of 3750 kg hm^−2^ lime. The content of free amino acid with 0.2 mol L^−1^ oxalic acid spraying treatment decreased by 49.76% compared with that without oxalic acid spraying treatment. The content of free amino acid reached the highest under without oxalic acid spraying treatment, which was 2.09 mg g^−1^. The content of free amino acid (1.05 mg g^−1^) with 0.2 mol L^−1^ oxalic acid spraying treatment was the lowest (Fig. 4).

The content of proline in roots decreased with the increase of the amount of lime application and oxalic acid spraying treatment. There was no significant difference in the proline content of *P. notoginseng* root under non-lime application. The proline content decreased at first and then increased with the increase of oxalic acid spraying concentration and 750 or 2250 kg hm^−2^ lime application. The proline content with 0.2 mol L^−1^ oxalic acid spraying treatment was significantly higher than that of 0.1 mol L^−1^ oxalic acid spraying treatment, which increased by 19.52% and 44.33%, respectively. Under the application of 3750 kg hm^−2^ lime, the content of proline decreased significantly with the increase of oxalic acid spraying concentrations. The content of proline with 0.2 mol L^−1^ oxalic acid spraying decreased by 54.68% compared with that without oxalic acid. The content of proline was the lowest under 0.2 mol L^−1^ oxalic acid treatment, which was 11.37 μg g^−1^ (Fig. 4).

### Contents of saponins

The contents of *P. notoginseng* saponins was Rg1 > Rb1 > R1. The contents of the three saponins had no significant difference with increase of the concentrations of oxalic acid spraying and no application of lime (Table 4).

**Table 4:** Effects of foliar oxalate application on the percentages of three saponins in roots of *Panax notoginseng* under Cd stress.

Oxalic acid concentration (mol L^−1^)	R1 (%)				Rg1 (%)			
	Lime application (kg hm^−2^)				Lime application (kg hm^−2^)			
	0	750	2250	3750	0	750	2250	3750
0.00	0.59 ± 0.04aB	0.74 ± 0.11aAB	0.69 ± 0.12aAB	0.88 ± 0.11aA	3.90 ± 0.30aA	3.99 ± 0.03bA	3.67 ± 0.20aA	3.23 ± 0.28aB
0.10	0.65 ± 0.07aA	0.70 ± 0.07abA	0.76 ± 0.14aA	0.57 ± 0.24abA	3.62 ± 0.15aB	4.35 ± 0.16aA	3.51 ± 0.29aB	2.58 ± 0.24bC
0.20	0.73 ± 0.05aA	0.48 ± 0.11bAB	0.63 ± 0.12aAB	0.41 ± 0.17bB	3.27 ± 0.58aB	4.08 ± 0.08bA	3.46 ± 0.15aAB	2.44 ± 0.12bC

Oxalic acid concentration (mol L^−1^)	Rb1 (%)				SPN (%)			
	Lime application (kg hm^−2^)				Lime application (kg hm^−2^)			
	0	750	2250	3750	0	750	2250	3750
0.00	1.56 ± 0.12aB	1.66 ± 0.12aAB	1.98 ± 0.20bAB	2.06 ± 0.30aA	6.05 ± 0.44aA	6.38 ± 0.05aA	6.33 ± 0.18bA	6.17 ± 0.43aA
0.10	1.54 ± 0.19aC	1.80 ± 0.09abBC	3.46 ± 0.12aA	2.12 ± 0.34aB	5.82 ± 0.17aC	6.82 ± 0.14aB	7.73 ± 0.44aA	5.27 ± 0.04ab
0.20	1.53 ± 0.13aA	2.40 ± 0.42aA	2.35 ± 0.29bA	1.76 ± 0.65aA	5.53 ± 0.58aAB	6.97 ± 0.53aA	6.44 ± 0.24bA	4.60 ± 0.91bB

Date were means ± SD. The same saponin with different lowercase letters and the same amount of lime were significantly different between the treatments (*P* < 0.05), and the same saponin with the same uppercase letter indicated that there were significant differences between the treatments under the same oxalic acid (*P* < 0.05).

The contents of R1 with 0.2 mol L^−1^ oxalic acid spraying was significantly lower than that without oxalic acid spraying and rates of 750 or 3750 kg hm^−2^ lime application. Under the concentration of 0 or 0.1 mol L^−1^ oxalic acid spraying, there was no significant difference in contents of R1 with increase of rates of lime application. Under the concentration of 0.2 mol L^−1^ oxalic acid spraying, the contents of R1 with 3750 kg hm^−2^ lime was significantly lower 43.84% than that without lime application (Table 4).

The contents of Rg1 increased at first and then decreased with the increase of oxalic acid spraying concentrations and 750 kg hm^−2^ lime application. Under the application rates of 2250 or 3750 kg hm^−2^ lime, the contents of Rg1 decreased with the increase of oxalic acid spraying concentration. With the same concentration of oxalic acid spraying, the Rg1 content increased at first and then decreased with the increase of lime application rates. Compared with the control, except that the Rg1 content with three concentrations of oxalic acid spraying and 750 kg hm^−2^ lime was higher than that of the control, the contents of Rg1 in the roots of *P. notoginseng* under other treatments was lower than that of the control. The Rg1 content was the highest with 750 kg hm^−2^ lime and 0.1 mol L^−1^ oxalic acid spraying treatment, which was higher 11.54% than that of the control (Table 4).

The contents of Rb1 increased first and then decreased with the increase of oxalic acid spraying concentration and 2250 kg hm^−2^ lime application. The content of Rb1 with 0.1 mol L^−1^ oxalic acid spraying reached the maximum value of 3.46%, which was higher 74.75% than that without oxalic acid spraying treatment. Under other lime application treatments, there was no significant difference among different oxalic acid spraying concentrations. With 0.1 and 0.2 mol L^−1^ oxalic acid spraying treatments, the contents of Rb1 decreased at first and then decreased with the increase of lime application rates (Table 4).

### Contents of flavonoids

**Figure 5:**
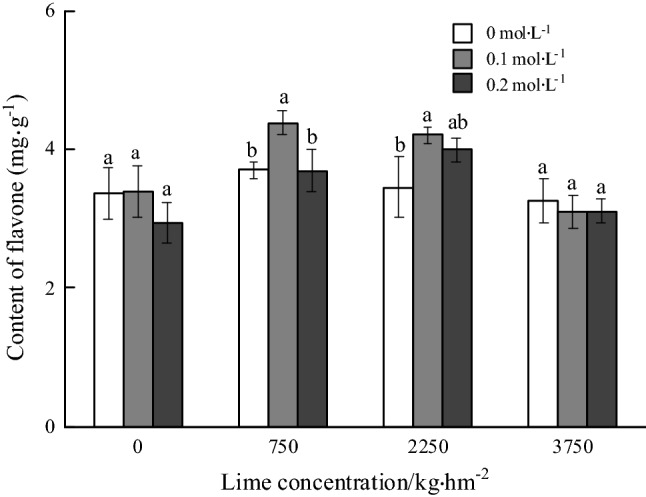
Effects of foliar spraying of oxalate on the contents of flavonoids in roots of *Panax notoginseng* under Cd stress.

With the same concentration of oxalic acid spraying, the content of flavonoids increased at first and then decreased with the increase of the amounts of lime application. There was no significant difference in the content of flavonoids under different concentrations of oxalic acid spraying without the application of lime or 3750 kg hm^−2^ lime. Under 750 and 2250 kg hm^−2^ lime application, the content of flavonoids increased at first and then decreased with the increase of the concentration of oxalic acid spraying. Under the treatment of 750 kg hm^−2^ application and 0.1 mol L^−1^ oxalic acid spraying, the content of flavonoids was the highest, which was 4.38 mg g^−1^, which was higher 18.38% than that of the same rate of lime application and without spraying oxalic acid. The content of flavonoids with 0.1 mol L^−1^ oxalic acid spraying treatment increased by 21.74% compared with that without oxalic acid spraying treatment and 2250 kg hm^−2^ lime application (Fig. 5).

Bivariate analysis showed that the content of soluble sugar in *P. notoginseng* root was significantly relationship with the amount of lime application and the concentration of oxalic acid spraying. The content of soluble protein in root was significantly relationship with lime application rates, both of lime and oxalic acid. The contents of free amino acid and proline in roots were significantly relationship with lime application rates, oxalic acid spraying concentrations, both of lime and oxalic acid (Table 5).

**Table 5:** Variance analysis of the effects of oxalic acid, calcium and cadmium on the contents of multiple medicinal ingredients in the roots of *Panax notoginseng* (F value).

Treatments	Soluble sugar	Free amino acid	Proline acid	Soluble protein	R1	Rg1	Rb1	SPN	Flavonoids
Limes	26.63**	18.60**	90.08**	70.03**	0.41	30.12**	13.13**	17.50**	13.41**
Oxalic acid	9.00**	13.49**	16.78**	8.59**	3.61*	4.53**	4.01*	3.22	3.73*
Lime × Oxalic acid	10.27**	20.21**	6.97**	13.05**	2.59*	1.82	4.77**	4.13**	2.09

The content of R1 in the root of *P. notoginseng* was significantly relationship with oxalic acid spraying concentrations, lime application rates, both of lime and oxalic acid. The content of flavonoids was significantly relationship with oxalic acid spraying concentrations, lime application rates.

## Discussion

Many amendments have been used to reduce plant Cd content by immobilizing Cd in soil, such as lime and oxalic acid^30^. Lime is widely applied as a soil amendment to reduce the Cd content in crop^31^. Liang et al.^32^ reported that oxalic acid can also be used as the remediation of heavy metal contaminated soil. After adding different concentrations of oxalic acid to the polluted soil, the organic matter of the soil increased, the cation exchange capacity decreased and the pH increased^33^. Oxalic acid can also react with metal ions in soil. Under Cd stress, the content of Cd in *P. notoginseng* increased significantly compared with the control. However, it is significantly reduced under the application of lime. In this study, the content of Cd in the root reached the national standard (the Cd limit was Cd ≤ 0.5 mg kg^−1^, AQSIQ, GB/T 19086-2008^34^) when 750 kg hm^−2^ lime was applied, and the effect was the best when 2250 kg hm^−2^ lime was applied. The application of lime brought a lot of competition sites between Ca^2+^ and Cd^2+^ in soil, and the addition of oxalic acid could reduce the Cd content in *P. notoginseng* roots. However, the content of Cd in the roots of *P. notoginseng* significantly decreased with the combination of lime and oxalic acid, reach the national standard. Ca^2+^ in soil was adsorbed to the root surface through mass flow process, and may be absorbed into root cells through calcium channel (Ca^2+^ channels), calcium pump (Ca^2+^-AT-Pase) and Ca^2+^/H^+^ antiporter, and then transported horizontally to root xylem^23^. There was a significant negative correlation between Ca content and Cd content in roots (*P* < 0. 05). Cd content decreased with the increase of Ca content, which was consistent with the view of antagonistic effect of Ca and Cd. Variance analysis showed that the amount of lime significantly affected the content of Ca in the roots of *P. notoginseng*. Pongrac et al.^35^ reported that Cd associates with oxalate in Ca oxalate crystals and competes with Ca. However, the regulation of Ca by oxalic acid was not significant. It indicated that the precipitation of Ca oxalate produced by oxalic acid and Ca^2+^ was not a simple precipitation, and the coprecipitation process may be controlled by multiple metabolic pathways.

Under Cd stress, a large number of reactive oxygen species (ROS) are formed in plants to destroy the structure of cell membrane^36^. The content of malondialdehyde MDA, can be used as an index to judge the level of ROS and the degree of plasma membrane damage in plants^37^. Antioxidant system is an important protective mechanism for scavenging reactive oxygen species^38^. The activities of antioxidant enzymes, include POD, SOD and CAT usually change in response to Cd stress. The results showed that MDA content was positively correlated with Cd concentration, indicating that the degree of plant membrane lipid peroxidation deepened with the increase of Cd concentration ^37^. This was consistent with the results of Ouyang et al.^39^. This study shows that MDA content was significantly affected by lime, oxalic acid, both of lime and oxalic acid. The MDA content of *P. notoginseng* decreased with 0.1 mol L^−1^ oxalic acid spraying, which was oxalic acid can reducing the bioavailability of Cd and the level of ROS in *P. notoginseng*. And antioxidant enzyme system is the site of plant detoxification function. SOD scavenges O^2−^ contents in plant cells to produce nontoxic O_2_, and low toxicity H_2_O_2_, POD and CAT scavenge plant tissue H_2_O_2_ and catalyze the decomposition of H_2_O_2_ into H_2_O. Based on iTRAQ proteome analysis, the protein expression level of SOD and PAL with lime application under Cd stress decreased and expression level of POD increased^40^. The activities of CAT, SOD and POD in the roots of *P. notoginseng* were significantly affected by the amount of oxalic acid and lime. The activities of SOD and CAT significantly increased with 0.1 mol L^−1^ oxalic acid spraying treatment, but had no obvious effect on regulating the activity of POD. It indicated that oxalic acid accelerated the decomposition of ROS under Cd stress, and completed the scavenging of H_2_O_2_ mainly by regulating the activity of CAT, which was similar to the results of Guo et al.^41^ on antioxidant enzymes in Potherb mustard (*Brassica juncea*, Coss.). The enzyme activities of antioxidant system and MDA contents with application of 750 kg hm^−2^ lime had similar effects with oxalic acid spraying. The result showed that oxalic acid spraying treatment can more effectively improve the activities of SOD and CAT and enhance the stress resistance of *P. notoginseng*. The activities of SOD and POD decreased with 0.2 mol L^−1^ oxalic acid and 3750 kg hm^−2^ lime treatment, indicating that excessive high concentrations of oxalic acid spraying and Ca^2+^ would be stress on plants, which was consistent with the study of Luo et al.^42^.

The medical components of *P. notoginseng* include soluble sugar, soluble protein, free amino acid, proline, flavonoids and saponins, which could response to environmental stress. Soluble sugar, soluble protein, free amino acid and proline are the main osmotic regulators, and flavonoids and saponins are the main secondary metabolites^24,43^. Plant cells will accumulate a large number of osmotic regulatory substances such as proline, soluble sugar and soluble protein to reduce cell osmotic potential and regulate plant physiological metabolism under stress^44^. Soluble protein is of an important osmotic regulator and nutrient. The accumulation of soluble protein content could improve the water retention capacity of cells and protect cell life substances and cytomembrance, so it is often used as one of the resistance indicators of plants^45^. In this experiment, the content of soluble protein in the roots of *P. notoginseng* was regulated by lime and oxalic acid, and there was a significant positive correlation between the content of soluble protein and the content of MDA. This indicated that the content of MDA in roots increased under Cd stress, and *P. notoginseng* could cope with stress by increasing the content of soluble protein. Proline plays an important role in resisting heavy metal toxicity. Exogenous proline treatment can reduce the inhibitory effect of Cd on poplar *PSII* reaction center activity, enhance leaf photosynthesis efficiency and improve plant stress resistance^29,46^. The content of free amino acid and proline decreased at first and then increased with the increase of oxalic acid concentration. The spraying of suitable exogenous oxalic acid concentrations increased the activity of antioxidant enzymes and changed the content of the main substances free amino acid and proline, which are important mechanisms for regulating the resistance of *P. notoginseng*.

Saponins and flavonoids are important active medical components of *P. notoginseng*. Saponins are a kind of glycosides formed by the condensation of steroidal or triterpene saponins with sugars or uronic acids. Liao et al.^47^ reported that Cd can reduced the content of saponins in *P. notoginseng*, mainly due to the down regulation of key enzyme genes *MVK*, *PMK* and *GGPS* of saponins metabolism under the influence of Cd stress. In this experiment, the contents of saponins of *P. notoginseng* was in order Rg1 > Rb1 > R1. In the case of non-lime application, the contents of saponins had no significant difference with the oxalic acid spraying. Under lime application and 0.2 mol L^−1^ oxalic acidspraying, the contents of saponins decreased significantly, which showed that high concentration of oxalic acid spraying decreased the yield of saponins. With the same the concentration of oxalic acid spraying, the contents of three saponins significantly increased with the increase of lime application rates. The contents of total saponins in the roots of *P. notoginseng* was significantly regulated by lime, and Ca^2+^ effectively promoted the yield of saponins. Based on iTRAQ proteome analysis, the protein expression levels of MVK, P450 and *β*-amyrin synthase, which were relative to saponins synthesis, decreased resulting in decrease 8.6–23.6% in saponins contents with lime application under Cd stress^48^. Based to the Pharmacopoeia of the People’s Republic of China, the total amount of notoginsenoside R1, ginsenoside Rg1 and ginsenoside Rb1 in dried *P. notoginseng* is more than 5.0% for high quality *P. notoginseng*. The contents of saponins met the standard value of high quality *P. notoginseng* saponins except for the treatment of 2250 kg hm^−2^ lime application and 0.2 mol L^−1^ oxalic acid spraying. Flavonoids exist in the form of bound (flavonoid glycosides) or free (flavonoid aglycone) in *P. notoginseng*. Zhang et al.^49^ reported that Cd stress effect *A. auriculiformis*. antioxidants, flavonoids and other corresponding gene abundance decreased. Therefore, Cd stress may reduces the activity of related enzymes in the process of flavonoid metabolism resulting in reduced flavonoid synthesis. It is consistent with the result that Cd stress will reduce the content of flavonoids in this study. And The expression levels of chalcone synthase (CHS) and ketone reductase (CHI) related to flavonoid metabolism were up-regulated and the flavonoid synthesis in plants was promoted by increasing the expression levels of flavonoid metabolism-related enzyme genes^25,50^. There was no significant difference in flavonoid contents among different concentrations of oxalic acid spraying except for 0 and 3750 kg hm^−2^ lime application. With the increase of oxalic acid spraying concentration, the contents of flavonoids increased at first and then decreased under 750 and 2250 kg hm^−2^ lime application, which showed that the regulatory effect of oxalic acid on flavonoids was limited. Oxalic acid not only increased the yield of flavonoids and affected the quality of *P. notoginseng*, but also improved the stress resistance to Cd.

**Figure 6:**
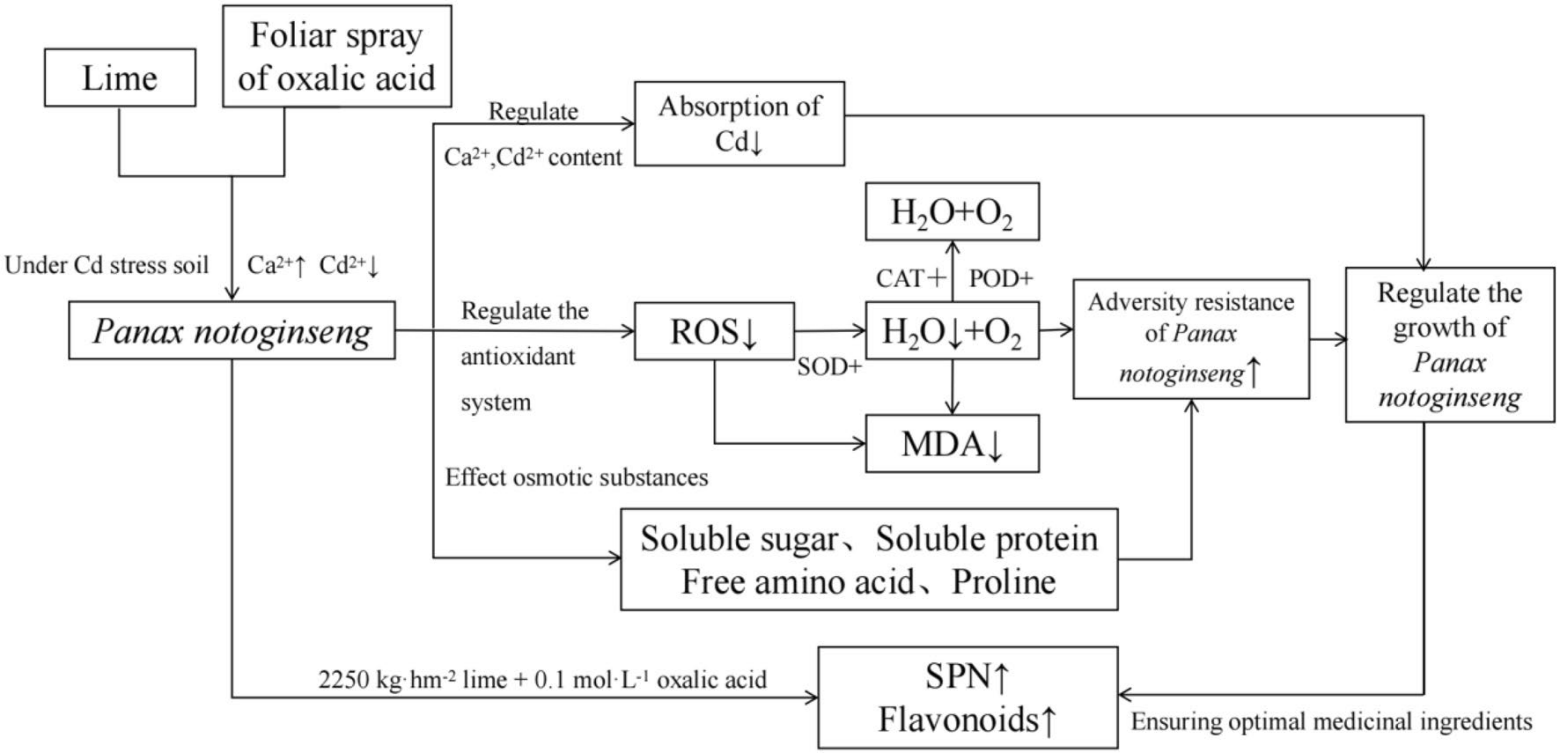
Effect of lime application and leaf spraying oxalic acid on resistance physiology of *Panax notoginseng*. *Notes* ↑ indicates improvement; ↓ indicates inhibiting effects.

In conclusion, the exogenous addition of lime and oxalic acid affected the physiological regulation of *P. notoginseng* through Ca and Cd interaction under Cd stress. Under 2250 kg hm^−2^ lime application, 0.1 mol L^−1^ oxalic acid treatment, Ca ions increased, Cd content decreased, MDA content was lower, antioxidant enzyme activities (POD, SOD, CAT) were all increased compared with the control treatment. Meanwhile, it was ensured that the SPN in the medicinal ingredients was the highest under this treatment, and the flavonoid content was also significantly increased compared with the control treatment (Fig. 6).

## Conclusion

The exogenous application of appropriate lime and oxalic acid increased antioxidant enzyme activity and reduced Cd content, enhancing the resistance of *P. notoginseng* under Cd stress. Lime application reduced soil uptake of Cd, increased POD activity, and decreased MDA content. Oxalic acid spraying significantly increased SOD and CAT activities and decreased MDA content. *P. notoginseng* mainly increased the contents of soluble sugar, free amino acid, proline and soluble protein under Cd stress. The resistance of *P. notoginseng* and the yield of saponins and flavonoids were significantly increased with moderate application of lime and oxalic acid. However, we found that excessive oxalic acid reduced the yield of *P. notoginseng* saponins and flavonoids, while Ca^2+^ effectively promoted the yield of total saponins. Therefore, it is recommended to apply 2250 kg hm^−2^ lime and spray 0.1 mol L^−1^ oxalic acid in order to ensure the content of Cd in *P. notoginseng* accords with the medicinal safety standards and to increase the yields of saponins and flavonoids.

## Data Availability

The data that support the findings of this study are available from the first author upon reasonable request. Experimental research and field studies on plants, including the collection of plant material, comply with relevant institutional, national, and international guidelines and legislation.
